# Prednisolone in Dogs—Plasma Exposure and White Blood Cell Response

**DOI:** 10.3389/fvets.2021.666219

**Published:** 2021-06-09

**Authors:** Carl Ekstrand, Helena Pettersson, Ronette Gehring, Mikael Hedeland, Sara Adolfsson, Inger Lilliehöök

**Affiliations:** ^1^Division of Pharmacology and Toxicology, Department of Biomedical Sciences and Veterinary Public Health, Swedish University of Agricultural Sciences, Uppsala, Sweden; ^2^Division of Clinical Pathology, Department of Clinical Sciences, Swedish University of Agricultural Sciences, Uppsala, Sweden; ^3^Clinical Pathology Laboratory, University Animal Hospital, Swedish University of Agricultural Sciences, Uppsala, Sweden; ^4^Division of Veterinary and Comparative Pharmacology, Department of Population Health Sciences, Utrecht University, Utrecht, Netherlands; ^5^Department of Chemistry, Environment and Feed Hygiene, National Veterinary Institute, Uppsala, Sweden; ^6^Department of Medicinal Chemistry, Uppsala University, Uppsala, Sweden

**Keywords:** canine, glucocorticoid, pharmacokinetics, pharmacodynamics, immune-suppression

## Abstract

Glucocorticoids such as prednisolone are commonly used in dogs but there is sparse quantitative pharmacokinetic and pharmacodynamic information of this drug in this species. The objective of this study was to quantitatively characterize the concentration-effect relationship for prednisolone in dogs on neutrophil and lymphocyte trafficking and cortisol suppression. Nine beagles, 2–12 years old and part of a group for teaching/research were used in a 4-way crossover experiment including two treatments, active or placebo, administered either *per os* (PO) or intravenously (IV). Plasma was analyzed for prednisolone and cortisol using ultra-high performance liquid chromatography – tandem mass spectrometry. Leucocyte counts were performed in whole blood. Data was then analyzed by non-linear mixed effect modeling to estimate pharmacokinetic and pharmacodynamic parameters. After administration of prednisolone sodium succinate IV, the typical value (between subject variation) for total body prednisolone clearance was 1,370 ml/h·kg (13.4%). The volumes of the central and peripheral compartment were 2,300 ml/kg (10.7%) and 600 ml/kg (16.0%), respectively. The terminal plasma half-life was 1.7 h. The prednisolone plasma concentration producing 50% of the maximum response was 10 ng/mL (90.3%), 22.5 ng/ml (52.3%) and 0.04 ng/mL (197.3%) for neutrophil, lymphocyte and cortisol response, respectively. The administered dose (1 mg/kg) increased neutrophil and decreased lymphocyte numbers but not over the entire dosage interval of 24 h, due to the short half-life. However, glucocorticoids have a wide range of responses. An anti-inflammatory response due to altered gene transcription might have a longer duration. Future studies on the anti-inflammatory potency together with data presented are needed to optimize future dosage recommendations in dogs.

## Introduction

Prednisolone is a glucocorticoid commonly used in dogs for its anti-inflammatory and immune-suppressive effects. Despite its wide use, sparse information is available about the pharmacokinetics (PK) and pharmacodynamics (PD) of prednisolone in dogs. More than 35 years ago, some pharmacokinetic reports were published (1–4). More recently, prednisolone concentration-time data have been made available but without thorough PK-analyses (5, 6). The PK studies cited above were performed on few dogs and since then technical improvements have made the analytical techniques for drug quantification in plasma more sensitive. A lower limit of quantification (LOQ) makes it possible to accurately measure drug concentrations for an extended time after administration. This allows for an increased understanding of the disposition of the drug during the terminal phase of the concentration-time curve, better informing the value of the PK-parameters, and the relationship between lower drug concentrations and effects. An example is the improved understanding of dexamethasone disposition in horses after improvements to the analytical method (7, 8). The increased analytical sensitivity used by Soma et al. (8) revealed a third compartment with considerably longer terminal half-life than reported by Toutain et al. (7), which is important to understand drug accumulation and duration of cortisol suppression. In addition, modern personal computers and software allows more sophisticated PK/PD analyses, so called non-linear mixed effects models or population analyses that not only model individual concentration time-courses but also variability in the data (9). These models were first used in data sets where a limited number of samples were collected from a large number of subjects, but they are also useful to analyse richer data sets from fewer individuals (10, 11). In humans, there is information available about the potency and efficacy of prednisolone (12, 13). In veterinary medicine, this information is missing. Additional PK/PD information is needed to identify appropriate starting doses and design better clinical trials for future optimized dosage regimens. Hence, the aim of this study was to characterize the dose-concentration-time and concentration-effect relationships of prednisolone in dogs.

## Materials and Methods

### Animals

The study was conducted in nine beagle dogs that were a part of a group for teaching and research and accustomed to handling and sampling (Table 1). The dogs were kept in their home environment. Before each treatment, an intravenous (IV) catheter was placed in the cephalic vein for blood sampling. Before IV treatment an additional IV catheter was placed in the saphenous vein and used only to administer the prednisolone. The dogs were housed in groups of two to four dogs for social and welfare reasons.

**Table 1:** Population characteristics, prednisolone doses and treatment order.

**Dog ID**	**Sex**	**Age (years)**	**Bodyweight (kg)**	**Dose IV (mg)**	**Dose PO (mg)**	**Order**
1	Female	12	14.5	14.5	15	BADC
2	Female	7	14.2	14.2	15	BADC
3	Female	10	15.1	15.1	15	BADC
4	Male	5	15.1	15.1	15	BADC
5	Male	7	14	14	15	BADC
6	Male	8	14.5	14.5	15	ABCD
7	Male	5	15	15	15	ABCD
8	Male	2	16.8	16.8	17.5	ABCD
9	Male	2	15.4	15.4	15	ABCD

*Order of treatments: A = Prednisolone IV, B = placebo IV, C = Prednisolone PO, D = placebo PO*.

### Housing and Feeding

The rooms were 20 m^2^ with raised platforms, toys and resting spaces. During daytime, the dogs were let outside in a pen with a doghouse. The dogs had access to a large exercise pen with grass and tunnels once a week. Dogs were fed nutritionally complete commercial feed (Hills pet nutrition, Langeskog, Denmark) twice daily. Water was available *ad libitum*.

### Experimental Design

Groups of dogs housed together were randomly assigned to one of four treatments; active (prednisolone) *per os* (PO), active intravenously (IV), placebo PO or placebo IV in a 4-way crossover experiment. The order of treatments for each dog are given in Table 1.

### Treatments

For the active treatments, prednisolone-sodiumsuccinate (Precortalon® aquosum, Biocodex AB, Kista, Sweden) at a concentration of 25 mg/mL was administered once IV and prednisolone tablets (Prednisolon Pfizer, Pfizer AB, Sollentuna, Sweden) once daily for ten consecutive days PO in the dogs' morning meal. The prednisolone doses were 0.97–1.07 mg/kg and precise dose for each dog are given in Table 1. For the placebo treatments, saline (Natriumklorid Fresenius Kabi 9 mg/ml, Fresenius Kabi AB, Uppsala, Sweden) was used IV and a morning meal without prednisolone tablets was used PO.

### Sample Collection

**Figure 1:**
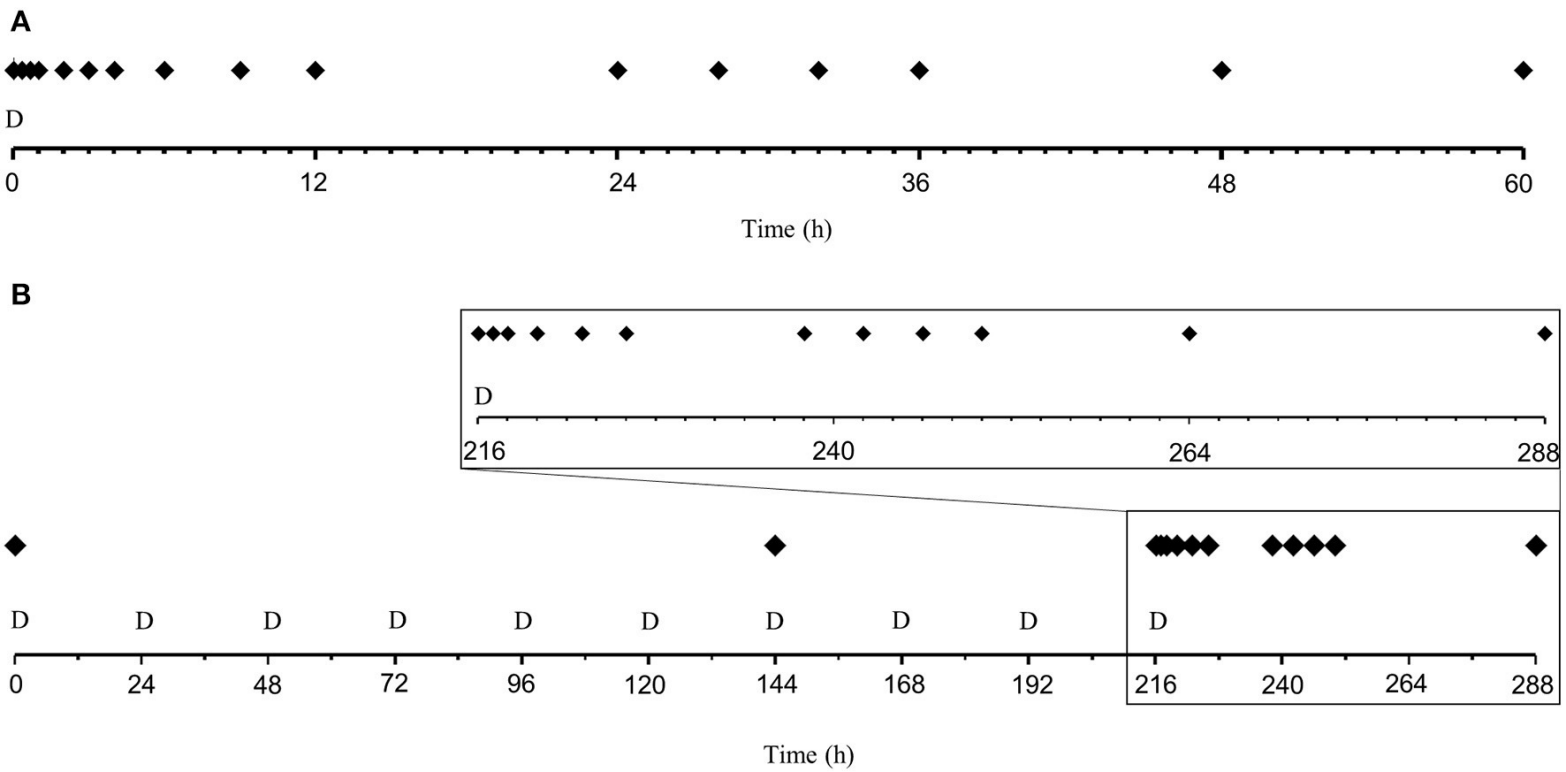
Schematic overview of the dose (D) and sampling (filled diamonds) regimen after administration of 1 mg/kg prednisolone succinate and placebo intravenously (IV, upper plot “**A**”) or ~1 mg/kg prednisolone or placebo once daily *per os* (PO, lower plot “**B**”) for 10 days to nine beagle dogs in a cross-over study. The magnification in the center show the 3 days following the last oral dose. **(A)** Dose and sampling regimen for IV administration. **(B)** Dose and sampling regimen for PO administration.

During the IV-treatment, blood was sampled before drug administration (0 h) and 0.33, 0.67, 1, 2, 3, 4, 6, 9, 12, 24, 28, 32, 36, 48, and 60 h after drug-administration. During PO-treatments blood was sampled before drug-administration at day 1 (0 h), 7 (144 h) and 10 (216 h). Additional blood was sampled 1, 2, 4, 7, 10, 22, 26, 30, 34, and 72 h after the last drug administration (217, 218, 220, 223, 226, 238, 242, 246, 250, and 288 h after the first dose). A schematic overview of the dosing and sampling protocol is shown in Figure 1. EDTA-coated tubes were used for all blood-samples. Blood for differential white blood cell count was transferred to new tubes before the rest of the blood was centrifuged at 2,100 *g*. The plasma was stored at −70°C pending prednisolone and cortisol analyses.

### White Blood Cell Differential Count

Total and differential white blood cell count was analyzed with canine settings with an ADVIA 2120 instrument with Multi Species Software (Siemens Healthcare, Dublin Ireland, L-00000062, Dec 20, 2018), a validated method more precise than manual counts (14).

### Analytical Method

The quantitative analyses of prednisolone and cortisol were carried out with ultra-high performance liquid chromatography – tandem mass spectrometry (UHPLC-MS/MS) at the National Veterinary Institute (SVA), Uppsala, Sweden. The reference compounds for prednisolone and cortisol and the internal standards [^2^H_8_]-prednisolon and [^2^H_7_]-hydrocortison were purchased from Toronto Research Chemicals (North York, ON, Canada). The sample preparation was performed as follows: to 100 μL of canine lithium heparinated plasma (study samples, calibrators or quality control samples), 100 μL of internal standard solution and 200 μL of trichloroacetic acid (20% v/v in water) was added for protein precipitation. The samples were then vortexed for 10 min and centrifuged at 10,000 *g* for 10 min. The supernatants were transferred to 96-well plates and 10 μL were injected into the UHPLC-MS/MS system consisting of an Acquity UPLC coupled to a Xevo TQ-S micro tandem quadrupole mass spectrometer equipped with an electrospray ionization interface operating in the positive mode, all from Waters Corp. (Milford, MA, USA). The analytical column was an Acquity UPLC BEH C18 (length 100 mm, inner diameter 2.1 mm, particle size 1.7 μm) held at 65°C. The mobile phase consisted of (A) 10 mM ammonium formate in water and (B) 0.1% formic acid in acetonitrile. Gradient elution was used: initially 23% B for 3.0 min, linear increase to 80% B for 0.5 min, constant at 80% B for 1.0 min, linear decrease to 23% B for 0.1 min, constant at 23% B for 0.9 min. The flow-rate was set to 400 μL/min. The electrospray parameters were: capillary voltage 0.50 kV and cone voltage 35 V. The desolvation and source block temperatures were 500 and 150°C, respectively. The desolvation gas flow-rate was 1,000 L/h. The quantification was carried out in the Selected Reaction Monitoring (SRM) mode with the collision cell filled with argon at 1.3 × 10^−5^ mBar. The SRM transitions used were *m/z* 361 → 147 for prednisolone (collision energy 25 eV) and *m/z* 369 → 150 for [^2^H_8_]-prednisolone (collision energy 25 eV), *m/z* 363 → 121 for cortisol (collision energy 25 eV) and *m/z* 369 → 100 for [^2^H_7_]-hydrocortison. The dwell time was 0.082 s. The calibration curves were constructed with the chromatographic peak area ratio (analyte/internal standard) as a function of the analyte concentration. Calibration and quality control samples for prednisolone were prepared by spiking reference compound to blank canine plasma. Calibration samples for cortisol were prepared by spiking reference compound to water, since this analyte is endogenous to the dog. Quality control samples of cortisol were prepared both by spiking reference compound to water and to canine plasma. The calibration functions were calculated by linear regression with a weighting factor of 1/x2 for both analytes.

### Pharmacokinetic and Pharmacodynamic Analyses

In order to obtain values for the area under the curve (AUC) to estimate the oral bioavailability of prednisolone, the plasma concentration-time data were subjected to non-compartmental analyses using WinNonlin 4.0.1 (Certara, St. Louis, Missouri, U.S.A). The PK data were then analyzed by fitting a compartmental model using the non-linear mixed effects (NLME) approach as implemented in the software Monolix2018R2 (Antony, France: Lixoft SAS, 2018). Visual inspection of diagnostic plots, the Akaike Information Criterion (AIC) and the Bayesian Information Criterion (BIC) were used to judge the best fit. Observations below the LOQ were treated as censored, i.e., any positive value below 0.05 ng/mL. The model parameters were the central (*V*_*c*_) and peripheral (*V*_*ti*_) volumes of distribution, plasma clearance (*Cl*) and inter-compartmental distribution clearance (*Cl*_*di*_), where *i* denotes the number of peripheral compartments needed to best describe the data. The parameter-values obtained after IV-analysis were then fixed so that the oral absorption rate constant (*k*_*a*_) and lag time in absorption (*t*_*lag*_) could be estimated from the PO data. For the PD analysis, the IV and PO PK models with parameter values fixed to the values estimated in the first step were used to simulate the prednisolone concentration-time course driving the PD-response. A turnover model was fitted to the PD-data (cortisol, lymphocyte- and neutrophil counts) obtained after both IV- and PO-administration of prednisone and saline (placebo). For cortisol, production was inhibited, for lymphocytes, loss was stimulated and for neutrophils, loss was inhibited. The model parameters were the maximum response (*I*_*max*_/*E*_*max*_), the concentration at 50% of maximum response (*IC*_50_/*EC*_50_), the sigmoidicity parameter (*n*), the baseline of the response (*R*_0_) and the fractional elimination rate of the response (*k*_*out*_).

The statistical model for between subject variability (BSV) was described by:

(1) $$\begin{array}{r} \theta_{i}=\theta_{tv}\cdot \exp\left(\eta_{i}\right) \end{array}$$

where θ_*i*_ is the value of the pharmacokinetic parameters in the *i*^th^ dog, θ_*tv*_ is the typical population value of *the parameter* and η_*i*_ is the deviation from the corresponding population value associated to the *i*^th^ dog (15). The exponential model assumes log-normal distribution of the parameters, i.e., that the distribution of the etas (η_*i*_) is normal in the log-domain, with a mean of 0 and the standard deviation of the random effects ω where η≈*N*[0, ω^2^]. The standard deviation of the random effects reported by Monolix® was then transformed to a coefficient of variation (CV%) using Equation (2):

(2) $$\begin{array}{r} CV\%=\sqrt{\exp\left(\omega^{2}\right)-1}\cdot 100 \end{array}$$

Shrinkage of the random effects (eta) toward the means was described as:

(3) $$\begin{array}{r} shrinkage=1-\frac{var\left(\eta_{r}\right)}{\omega^{2}} \end{array}$$

where *var*(η_*r*_) is the variance of the random effects. When shrinkage for eta were >30%, the random component was not considered robustly estimated. Sex, weight, and age were investigated as covariates. Impact on objective function values, parameter BSV and Monolix internal statistical functions were used in covariate evaluation.

## Results

### Analytical Method

The linear calibration ranges were 0.50–500 ng/mL for prednisolone and 0.27–543 ng/mL for cortisol. The precision (RSD%) was for prednisolone in the range of 1.6–11% and for cortisol 1.3–10%. The accuracy was for prednisolone in the range of 87–108% and for cortisol 87–104%.

### Model Evaluation

A two-compartment model was judged to be the best fit for the prednisolone PK-data. The between subject variability could be robustly estimated for all the PK parameters and for the potency (*IC*_50_), efficacy (*I*_*max*_) and the base line of response (*R*_0_) in the PD model. None of the covariates improved the model and these were therefore discarded. A schematic overview of the PK/PD model is shown in Figure 2. Diagnostic plots are available in Supplementary Figures 1–12.

**Figure 2:**
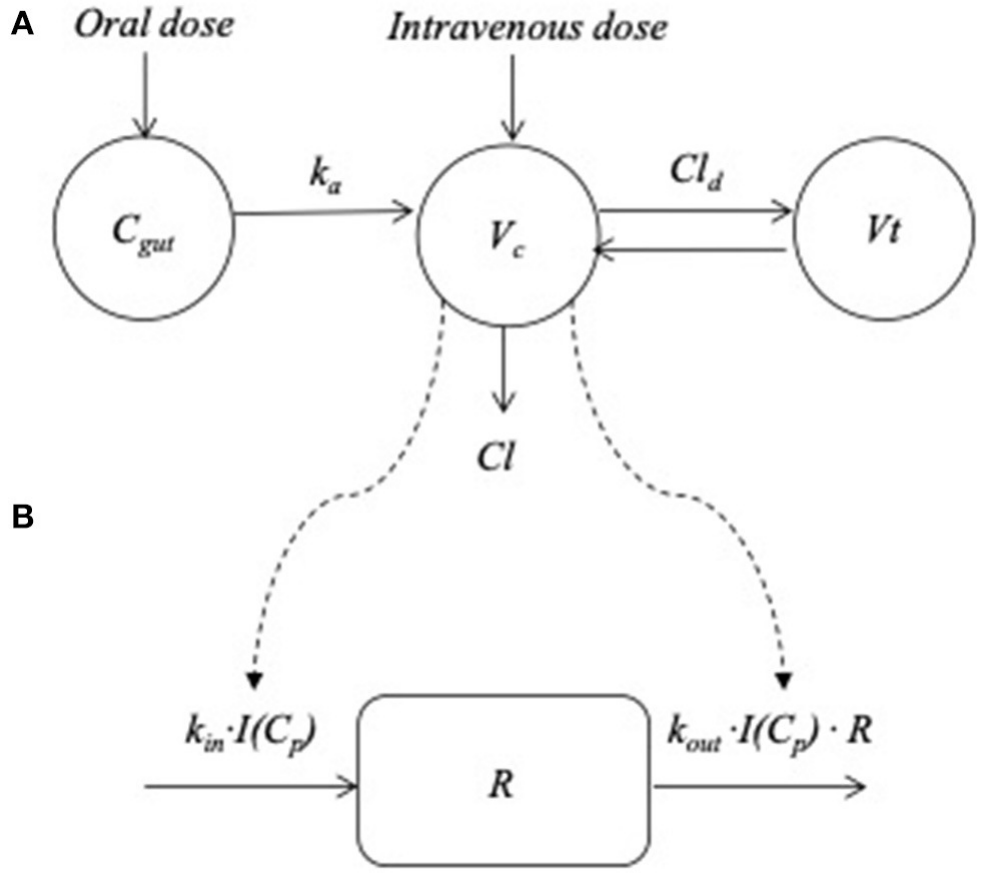
Overview of the pharmacokinetic and pharmacodynamics model. **(A)** The plasma absorption and disposition model where *C*_*gut*_* , k*_*a*_*, V*_*c*_, *V*_*t*_ , *Cl*, and *Cl*_*d*_ represent the drug disposed in the gut, the absorption rate constant from the gut to the central compartment, central and peripheral volume of distribution, clearance and inter-compartmental distribution parameter, respectively. **(B)** The turnover model describing the prednisolone induced changes in response (*R*). The plasma concentration (*C*_*p*_) was used to “drive” the drug mechanism function [*I* (*C*_*p*_)] acting on the turnover rate of response where *k*_*in*_(*t*)*, k*_*out*_ and *R* represent the turnover rate and the fractional turnover rate. For cortisol production of response was inhibited, for lymphocytes loss was stimulated and for neutrophils, loss of response was inhibited.

### Observed Plasma Prednisolone Concentrations

**Figure 3:**
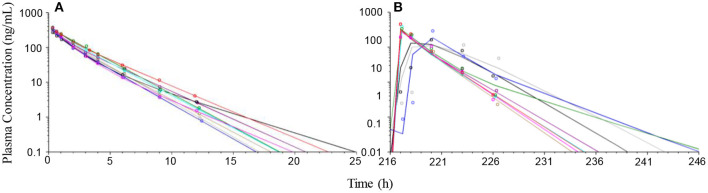
Observed (symbols) and model predicted (lines) prednisolone plasma concentration-time courses following administration of 1 mg/kg prednisolone succinate intravenously (left plot **A**) and after the last dose (216 h after first dose) of approximately 1 mg/kg for 10 consecutive daily oral doses (right plot **B**) to 9 beagle dogs. Twenty four hours after IV-administration or 24 h after the last dose *per os* (240 h after the first dose) prednisolone plasma concentration was below the lower limit of quantification (0.5 ng/mL) in all dogs.

After IV administration of 1 mg/kg prednisolone, plasma prednisolone concentrations decreased rapidly and observed concentrations were below LOQ (0.5 ng/mL) after 24 h in all dogs. Twelve hours after prednisolone administration, plasma prednisolone concentration ranged between 0.78 and 4.1 ng/mL (Figure 3). During oral treatment over a 10-day period, pre-administration prednisolone plasma concentrations only exceeded the LOQ once (0.79 ng/mL, day 7) in one dog. After the last dose, peak plasma concentrations were observed in eight of the dogs within 4 h after administration and one dog showing slower absorption and a peak concentration 7 h after administration (Figure 3). Ten hours after the last dose, plasma prednisolone concentrations ranged between 2.3 and 15.2 ng/mL in eight dogs. In one dog plasma prednisolone concentration was 42.6 ng/ml 10 h after last drug administration. This dog also showed the slowest absorption. At 22 h after last prednisolone administration plasma concentrations were below the LOQ in all dogs. Relative to plasma prednisolone exposure after IV administration, the PO bioavailability was median (range) 108% (87–118). The PO bioavailability could be biased by slow hydrolyses of the succinate ester and is hence a function of the bioavailability following IV administration (see section Discussion).

A two-compartment pharmacokinetic model fit the experimental prednisolone data collected after IV administration of prednisolone succinate well (Figure 3). The parameters were estimated with good precision (CV 4–19%). The model was then fitted to experimental prednisolone data collected after PO administration, but parameters could not be robustly estimated, most likely due to masking of the distribution phase by the absorption. The disposition parameters were therefore fixed so that only the absorption parameters needed to be estimated from the PO data. These parameters were estimated with acceptable to poor precision (CV 30–66%). The pharmacokinetic parameters and the corresponding BSV are shown in Table 2.

**Table 2:** Model estimated pharmacokinetic typical values, their corresponding relative standard deviation (CV%) and the between subject variation (BSV) and secondary pharmacokinetic parameters after intravenous (IV) administration of 1 mg/kg prednisolone succinate and administration of ~1 mg/kg prednisolone *per os* (PO).

**Model parameters**	**Unit**	**Typical value (CV%)**	**BSV (%)**
**IV**			
*V_*c*_*/F_IV_	mL/kg	2,300 (3.8)	10.7
*V_*t*_*/F_IV_	mL/kg	600 (5.7)	16.0
*Cl*/F_IV_	mL/h·kg	1,370 (4.5)	13.4
*Cl_*d*_*	mL/h·kg	530 (19.2)	58.2
**PO**			
*k_*a*_*	h^−1^	2.52 (65.9)	194.2
*t_*lag*_*	h	0.69 (30.1)	60.9
**Secondary parameters**	**Unit**		
α	h^−1^	1.3	–
β	h^−1^	0.4	–
*t_1/2α_*	h	0.5	–
*t_1/2β_*	h	1.7	–

*V_c_, V_t_, Cl, Cl_d_ k_a_, and t_lag_ represent central and peripheral volumes of distribution, clearance and inter-compartmental distribution parameter, the absorption rate constant from the gut to the central compartment and the lag-time, respectively. α, β, t_1/2α_, and t_1/2β_ represent the rate constant of the initial and terminal phase and the half-lives of the initial and terminal phase, respectively. F_IV_ indicate that the parameter values may be confounded of unknown bioavailability of prednisolone after IV administration of prednisolone succinate in dogs IV (see section Discussion)*.

### Neutrophil, Lymphocyte, and Cortisol Response

**Figure 4:**
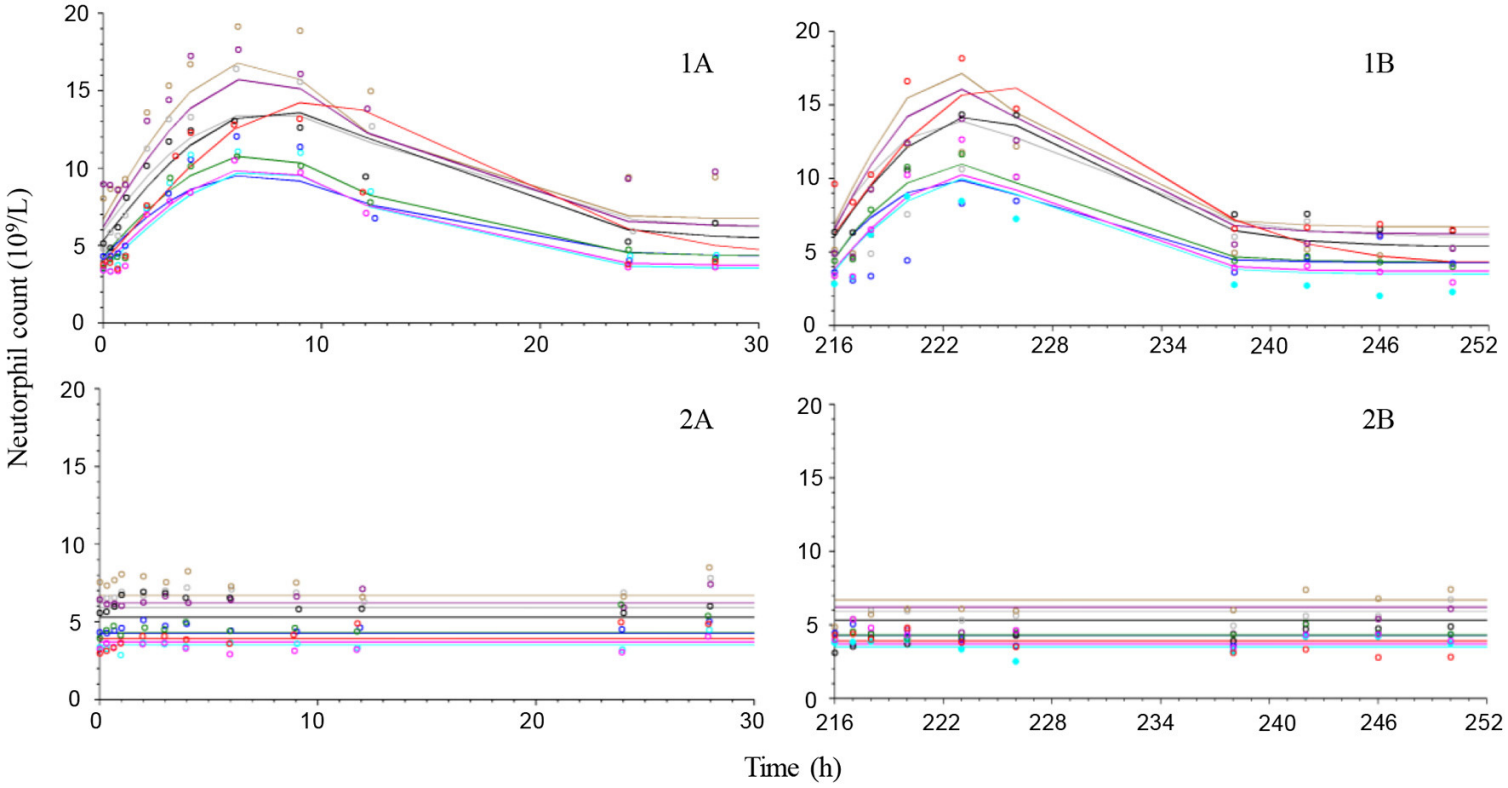
Observed (symbols) and model predicted (lines) neutrophil count-time courses following intravenous administration (left plots) of 1 mg/kg prednisolone succinate **(1A)** or saline **(2A)** and after the last oral (right plots) dose (216 h after first dose) of ~1 mg/kg for 10 consecutive daily doses orally **(1B)** or placebo **(2B)** to 9 beagle dogs.

**Figure 5:**
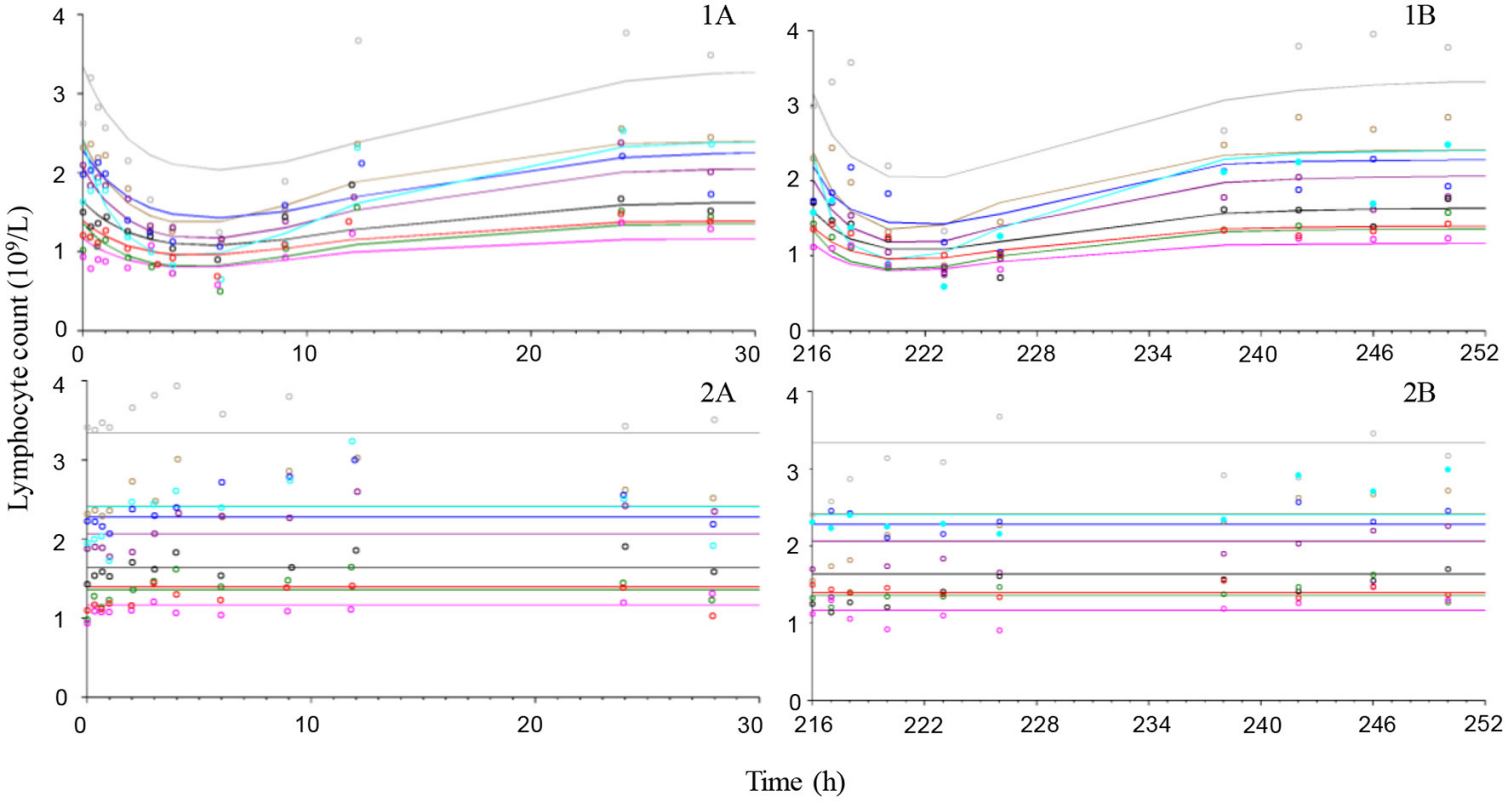
Observed (symbols) and model predicted (lines) lymphocyte count-time courses following intravenous administration (left plots) of 1 mg/kg prednisolone succinate **(1A)** or saline **(2A)** and after the last oral (right plots) dose (216 h after first dose) of ~1 mg/kg for 10 consecutive daily doses orally **(1B)** or placebo **(2B)** to 9 beagle dogs.

**Figure 6:**
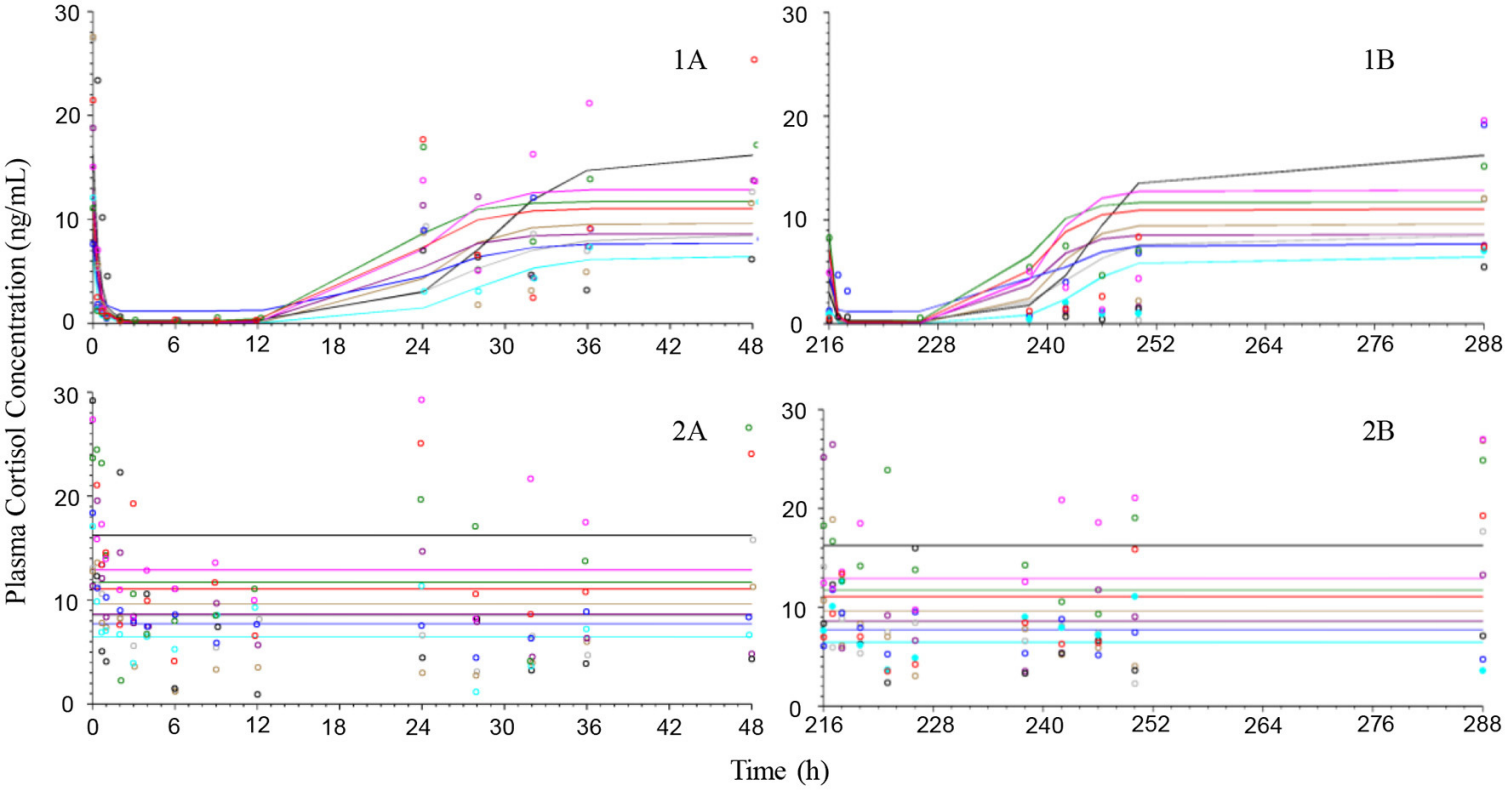
Observed (symbols) and model predicted (lines) cortisol plasma concentration-time courses following intravenous administration (left plots) of 1 mg/kg prednisolone succinate **(1A)** or saline **(2A)** and after the last oral (right plots) dose (216 h after first dose) of ~1 mg/kg for 10 consecutive daily doses orally **(1B)** or placebo **(2B)** to 9 beagle dogs.

Placebo treatment observations suggest that neutrophils and lymphocytes had limited variability compared to cortisol which was more variable both within and between animals (Figures 4–6). Exposure to prednisolone increased neutrophil counts (Figure 4) and decreased both lymphocyte counts (Figure 5) and cortisol plasma concentrations (Figure 6). All three response courses demonstrated an onset of response within 3 h, maximum response within 10 h and were estimated to be back to base line at 24–36 h. The turnover model described the observed data well and generated model parameters with acceptable to good precision. Pharmacodynamic parameter values, their precision and BSV are shown in Table 3.

**Table 3:** Model estimated pharmacodynamics typical values (t.v.), their corresponding relative standard deviation (CV%) and between subject variation (BSV) after intravenous administration of 1 mg/kg prednisolone succinate and ~1 mg/kg prednisolone *per os*.

**Parameter**	**Unit**	**Neutrophils**		**Lymphocytes**		**Cortisol**	
		**t.v. (CV%)**	**BSV (%)**	**t.v. (CV%)**	**BSV (%)**	**t.v. (CV%)**	**BSV (%)**
*IC_50_/EC_50_*	ng/mL	10 (36.9)	90.3	22.5 (32.6)	52.3	0.04 (50.1)	197.3
*I_*max*_/E_*max*_*	–	1 (5.5)	5.0	1 (14.4)	35.0	1 (1.6)	4.8
*n*	–	0.66 (2.2)	–	1 (0.07)	–	1 (0.02)	–
*R_0_*	10^9^/L^1^ ng/mL^2^	4.8^1^ (7.7)	23.1	1.9^1^ (10.8)	33.4	10^2^ (9.8)	28.2
*k_*out*_*	1/h	0.382 (4.2)	–	0.232 (3.93)	–	2.4 (4.4)	–

*IC_50_/EC_50_, I_max_/E_max_, n, R_0_, and k_out_ are the potency value, efficacy value, sigmoidicity parameter, baseline of response, and elimination-rate constant of response, respectively. ^1^Unit for Neutrophil/Lymphocyte cell counts. ^2^Unit for cortisol concentration*.

There were two missing values in neutrophil and lymphocyte response because one sample had clotted and one sample was lost because of technical problems. The missing values were both from active IV treatment and from two different dogs, 32 and 48 h after administration, respectively.

## Discussion

The dosage regimen (dose and dose interval) is one corner stone in pharmacological therapy. The PK (absorption, distribution, and elimination) of a drug combined with toxicology data and the turnover of the PD response decide the therapeutic window and the dosage regimen. This study describes the plasma exposure, PK and PD of prednisolone in dogs using neutrophil and lymphocyte cell counts as response to prednisolone exposure which provide additional evidence and input to future studies using clinical outcome or more relevant inflammatory/immunosuppressive biomarkers to optimize pharmacotherapy.

Prednisolone exposure after 1 mg/kg caused increased neutrophil numbers and decreased lymphocyte numbers that returned to baseline within 24 h from prednisolone administration. The return to baseline was due to the short half-life of prednisolone in dogs, resulting in plasma concentrations falling below effective concentrations values within hours, and the relatively rapid turnover and migration of these white blood cells (16–18). The 1 mg/kg dose is within the clinically established dose-range (0.5–4 mg/kg) for prednisolone. However, if treatment is to be continued for longer periods of time, the lowest effective dose should be used to minimize adverse effects. A dose of 0.5 mg/kg every second day is considered clinically effective in some cases. Considering the short terminal half-life (1.7 h) and the high *IC*_50_-values (10–22.5 ng/mL), concentrations above those *IC*_50_-values were maintained <24 h in plasma following oral administration of 1 mg/kg prednisolone to beagle dogs.

It is established that the clinical efficacy and half-life of response of corticosteroids is longer than the pharmacokinetic half-life for glucocorticoids (19). This is likely due to modulation of the transcription of genes encoding for inflammatory modulators that have a slower turnover than the biomarkers measured in this study. The immunosuppressive (and anti-inflammatory) responses to glucocorticoids are numerous (20). For instance, glucocorticoids induce synthesis of the anti-inflammatory protein Annexin A1 (21). Annexin A1 is stored in the cytoplasm and must be degraded before its effect disappears. Possibly prednisolone affects some of these biomarkers with a higher potency increasing the time in the therapeutic window. Both of these factors could explain the clinical effect that is longer than the duration of response reported here. In addition, low prednisolone doses are usually used against mild and chronic symptoms. Possibly, the short exposure inhibits the stimulatory processes within the turnover of low-grade inflammatory processes and thereby hinders development of clinical signs which could explain why the dose 0.5 mg/kg every second day can be clinically efficient. However, this hypothesis remains to be verified in future studies.

The previously presented pharmacokinetic parameter estimates for clearance and bioavailability are lower than their corresponding typical values in this study (1–3). Prednisolone is poorly soluble in water so esters (e.g., prednisolone phosphate and prednisolone succinate) are formulated and used for IV administration. Both prednisone phosphate and prednisolone succinate are prodrugs that need to be bio-converted (i.e., hydrolysed) before their active compound prednisolone becomes systemically available. Prednisolone phosphate is rapidly converted to prednisolone in dogs (22) which was interpreted as the entire prednisolone dose was made systemically available. The succinate ester has also been shown to be rapidly hydrolysed *in vivo* in rabbits but not *in vitro* in plasma or whole blood indicating that the active compound probably becomes systemically available due to organ clearance (23). In dogs, however, the fraction of the dose methylprednisolone made systemically available was 43% after IV administration of methylprednisolone succinate (24). Toutain et al. (24) argues that this species difference is due to a genetic predisposition for over-expression of carboxylases in the rabbit strain used in (23). If the results in (24) is also true for prednisolone succinate the results from the PK analyses presented here should be interpreted with great caution for other types of prednisolone esters. The PK-part of the study assumed that the dose administered IV was made 100% systemically available and used for estimation of both bioavailability and clearance. The PK model was successfully fitted to experimental data with good to acceptable precision and described the concentration-time course adequately. The potentially low systemic availability of prednisolone after IV administration of prednisolone warrants further attention in relation to PK-model output. If the bioavailability of prednisolone in dogs is similar to that of methylprednisolone in (24), it could explain the difference in bioavailability and mean clearance values reported after IV administration of prednisolone phosphate (1–3). It should be noted that the range of clearance values within the previously studied populations in (1–3) overlap the individual parameter range for clearance in this study. It cannot be excluded that the differences in results could be due to between study variability. Nevertheless, the fraction prednisolone made systemically available after IV administration of prednisolone phosphate and prednisolone succinate warrants future attention. Until then, the pharmacokinetic model parameter values reported in this study should be considered specific for the succinate formulation and only be extrapolated to formulations containing other salts cautiously. The terminal half-life, which is an important clinical parameter since it is used for estimation of dosage interval, depends on the rate of elimination which in turn shapes the slope of the terminal phase of the concentration-time course. The model was adequately fitted to experimental observations, mimicked the slope and therefore the half-life should be considered reliable. The half-lives were also confirmed by visual inspection of experimental data.

The major reason to perform a PK analyses on prednisolone data was to derive a PK-profile that then later was used to “drive” the PD-model. The predicted time-courses fitted experimental data well and PK-analyses were functional in this aspect (data not shown). Consequently, the PD-parameters for increased neutrophil numbers, decreased lymphocyte numbers and cortisol suppression deserve attention. These are known glucocorticoid responses (25–28). In other species white blood cell counts and cortisol suppression has also been used to derive quantitative PD-data and estimate the magnitude and duration of glucocorticoid response (12, 29, 30). The pharmacodynamics model was fitted to experimental data, the predicted time courses mimicked data well and the model parameters were estimated with good to acceptable precision. In order to maintain prednisolone plasma concentration above the potency value the entire dosage interval, an increase of the daily dose 1 mg/kg and more frequent dosing is necessary. If change in white blood cell counts are suitable surrogate markers for immunosuppressive responses to prednisolone, these results strengthen the empirical initial prednisolone daily dose for immunosuppression (2–4 mg/kg). It is, however, known that individual patients respond to lower doses (31, 32). Doses should, therefore, be individually tapered down to achieve the lowest possible clinically effective prednisolone dose for a specific patient. Hence, the results presented in this study are more useful as input in the design of clinical studies than for individual dose-recommendations. For example, Monte Carlo simulations can be performed with the model to predict the probability of favorable clinical outcomes for a given dosage regimen in a target population (33). These predictions can then be compared with experimental outcomes as part of the learn-confirm cycle of developing safe and effective veterinary therapies. It should also be noted that this study used Beagle dogs only. Inter-breed differences in PK and PD might occur (34) and the results from this study should be interpreted with this in mind. In the future, this model can be validated and refined with data from studies in other breeds to quantify any possible variability in PK and PD parameter values.

The BSV for the PK-parameters was low for parameters estimated with good precision. The parameters *k*_*a*_ and *t*_*lag*_ both had low precision and high BSV, which most probably was due to limited experimental data in the absorption phase. However, also the potency value (*IC*_50_*/EC*_50_-value) BSV was quite substantial in this study with greatest values for cortisol response. It is known that PD usually show larger variability between individuals than PK (35). To the best of our knowledge no BSV-values are published for glucocorticoid responses in dogs. In the horse, the BSV for cortisol suppression after dexamethasone exposure was up to 80% (36, 37), which is comparable with the BSV for neutrophil and lymphocyte *IC*_50_–values but lower than the variability on cortisol response potency in this study. The reason behind this variation is not fully known but sex and age has been shown to be sources of variation in cortisol concentration in dogs (38, 39). In the present study, age and sex were evaluated as covariates but could not explain the large BSV. Possibly, a larger study population would increase the chances in explaining BSV variation due to age and sex. Differences between species, substances and study design could provide additional possible explanations to the relatively high BSV.

In conclusion: Prednisolone is cleared rapidly which results in a short terminal half-life. After PO administration, maximum concentrations were observed within 1 h in most of the dogs. The administered dose (1 mg/kg) caused increased neutrophil numbers and decreased lymphocyte numbers but did not result in effective plasma concentration over the entire dosage interval of 24 h. However, glucocorticoids have a wide range of responses and e.g., anti-inflammatory responses due to altered gene transcription might have longer duration of effects. Future studies on the anti-inflammatory potency of prednisolone together with data presented here could possibly optimize future dosage recommendations in dogs.

## Data Availability Statement

The raw data supporting the conclusions of this article will be made available by the authors, without undue reservation.

## Ethics Statement

The animal study was reviewed and approved by the Animal Ethics Committee, Uppsala, Sweden (5.8.18-07216/2017).

## Author Contributions

CE, HP, SA, and IL planned and executed the experiment. MH and IL analyzed for prednisolone, cortisol, and performed cell counts. CE and RG performed PK/PD analyses. CE drafted the manuscript with assistance from RG. All authors revised and approved the final version of the manuscript.

## Conflict of Interest

The authors declare that the research was conducted in the absence of any commercial or financial relationships that could be construed as a potential conflict of interest.
