# Supplementary material for: Prednisolone in Dogs—Plasma Exposure and White Blood Cell Response
Source: Front Vet Sci. 2021 Jun 9;8:666219. doi: 10.3389/fvets.2021.666219 (PMC8219870; doi:10.3389/fvets.2021.666219)
Supplement: Supplementary file 1 [file Data_Sheet_1.PDF]

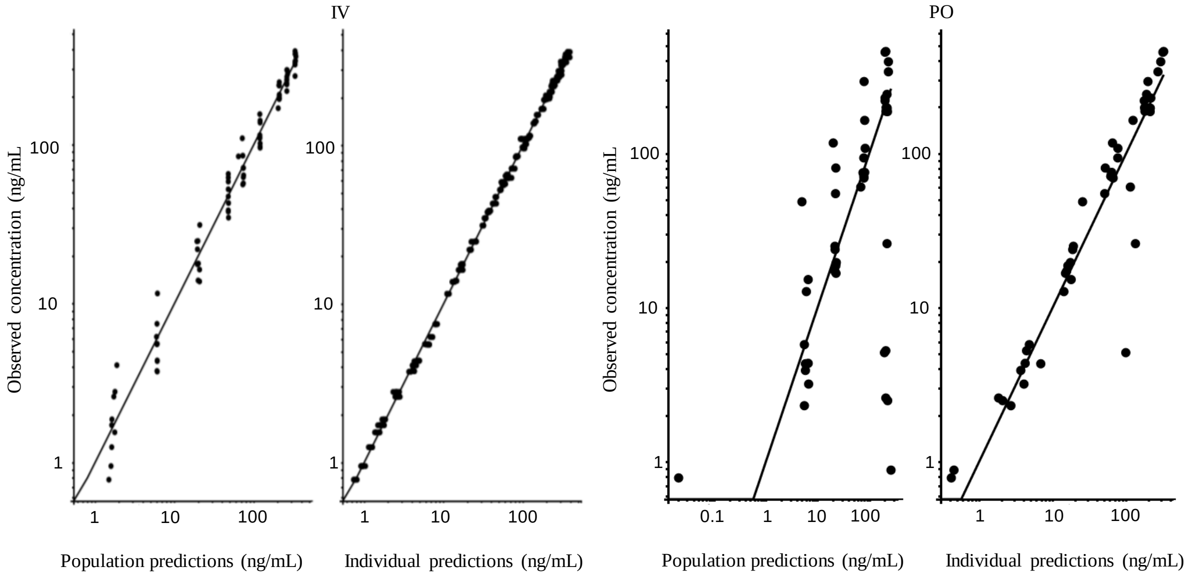

Fig S1. Observed vs Predicted prednisolone plasma concentration splitted by route of administration following 1 mg/kg prednisolone administration as either a bolus dose intravenously (IV, left) and after the last of ten daily 1 mg/kg dose *per os* (PO, right) to 9 beagle dogs.

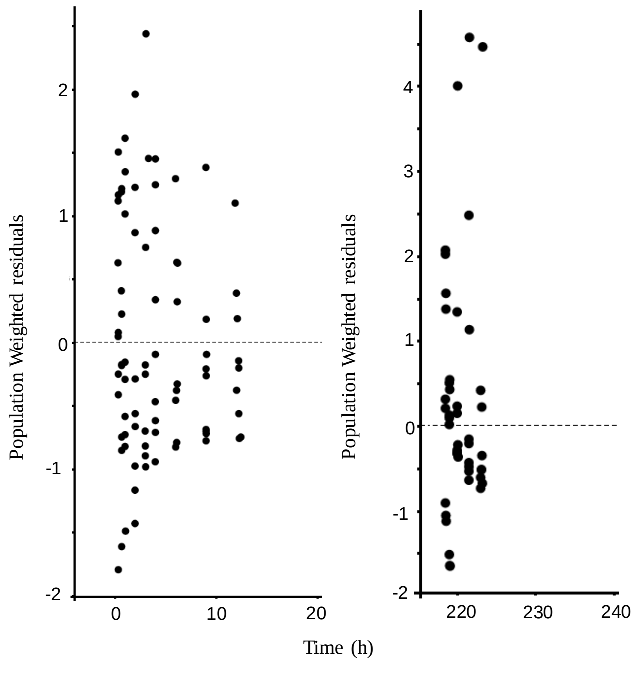

Fig S2. Weighted residuals over time for prednisolone plasma concentration splitted by route of administration following 1 mg/kg prednisolone administration as either a bolus dose intravenously (IV, left) and after the last of 10 daily doses of 1 mg/kg administered *per os* (PO, right) to 9 beagle dogs. Last oral dose was administered at 216 h.

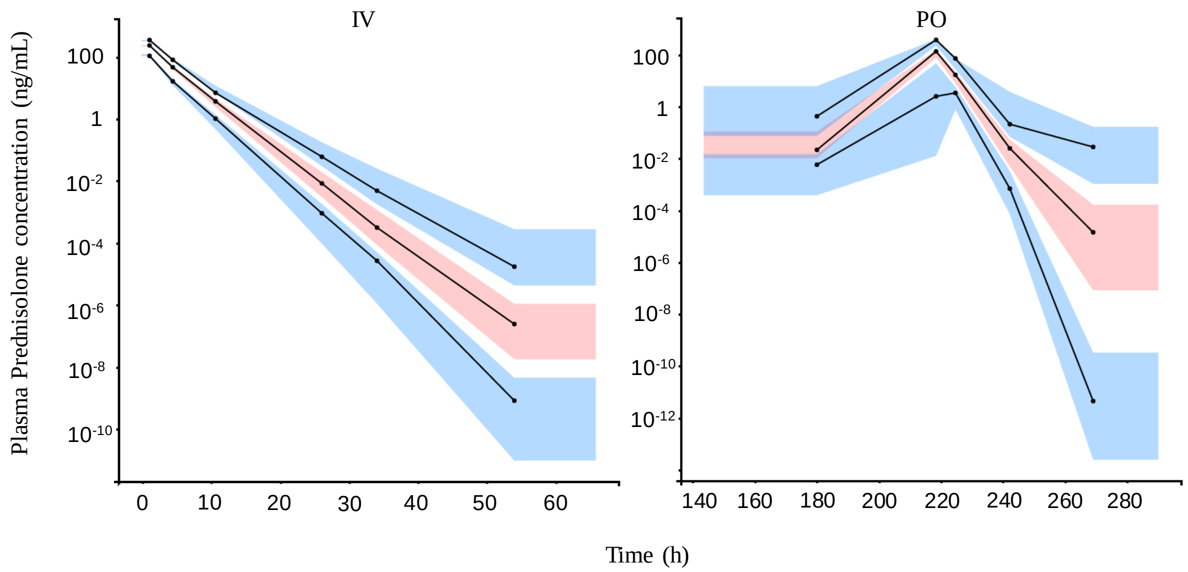

Fig S3 Visual predictive check (VPC) splitted by route of administration showing the empirical 10th, 50th and 90th percentiles and corresponding prediction interval for prednisolone plasma concentration following 1 mg/kg prednisolone and control administered intravenously (IV, left) and after 10 daily doses of 1 mg/kg administered *per os* (PO, right) to 9 beagle dogs. Last dose PO was administered at 216 h.

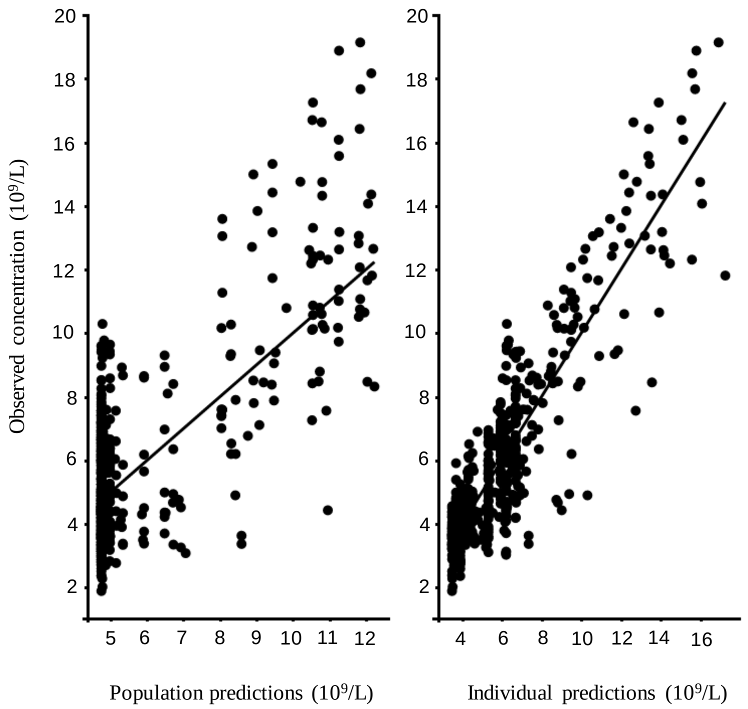

Fig S4. Observed vs predicted neutrophil whole blood cell count following a 1 mg/kg prednisolone administration intravenously (IV, left) and after 10 daily doses of 1 mg/kg administered *per os* (PO, right) to 9 beagle dogs.

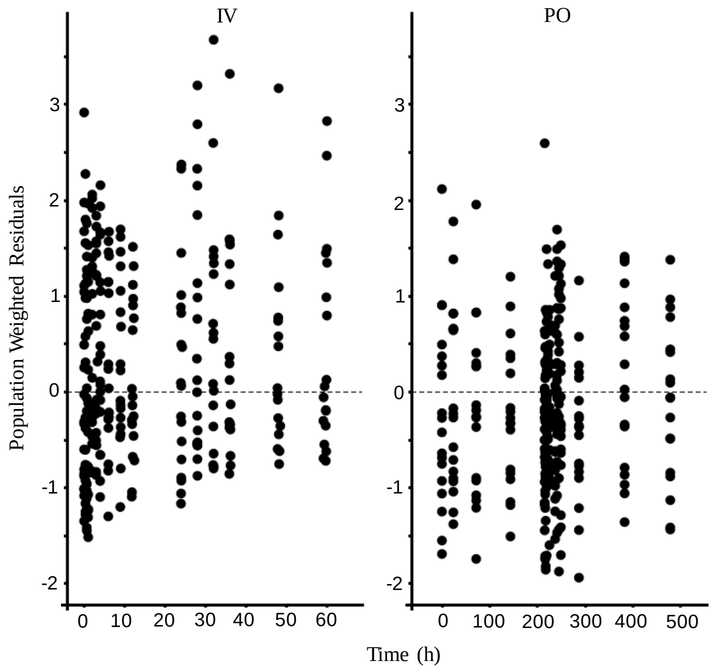

Fig S5. Weighted residuals over time for neutrophil whole blood cell count splitted by route of administration following 1 mg/kg prednisolone and control administered either as bolus dose intravenously (IV, left) and after 10 daily doses of 1 mg/kg administered *per os* (PO, right) to 9 beagle dogs. Last oral dose was administered at 216 h.

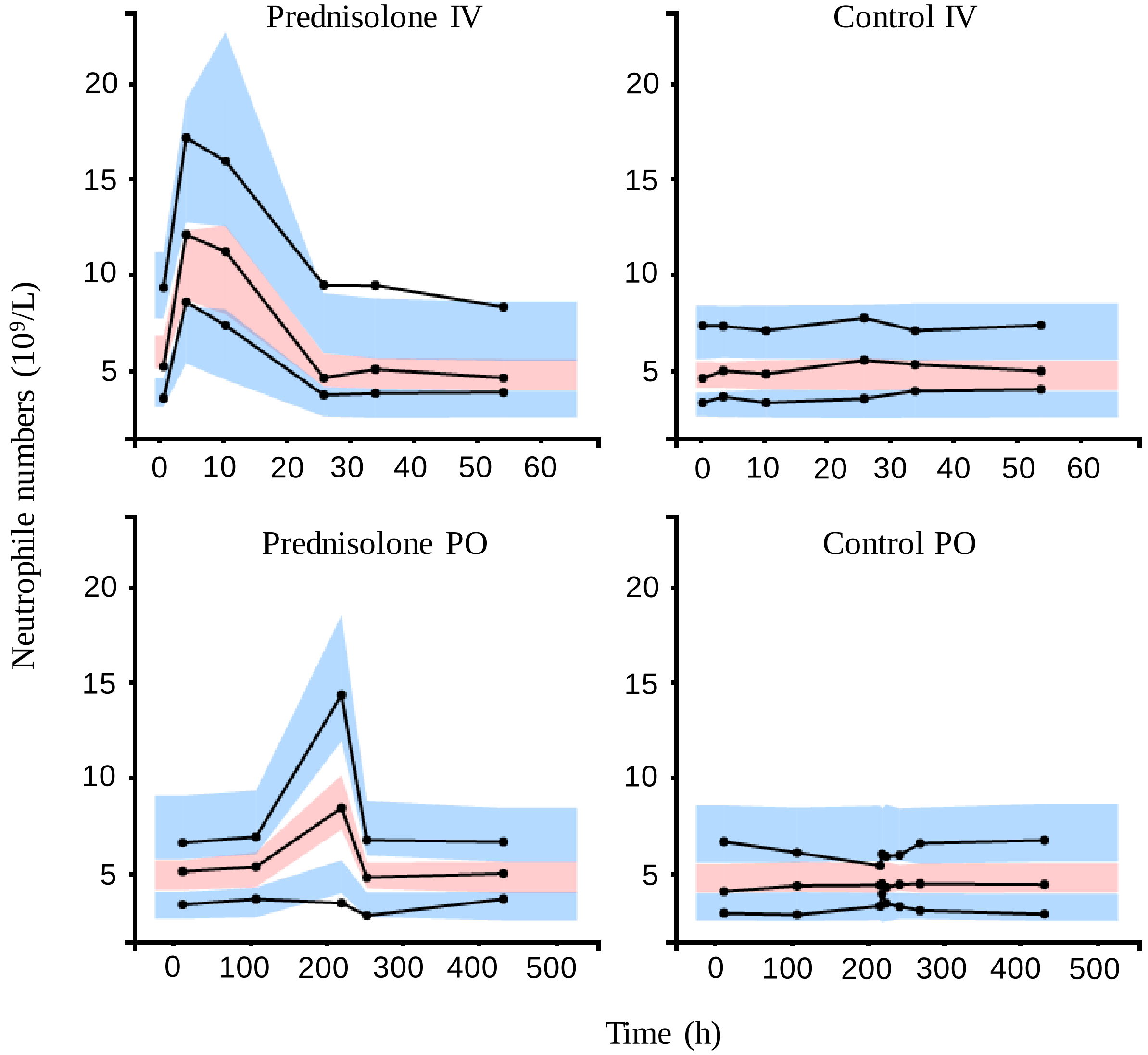

Fig 6. Visual predictive check (VPC) splitted by route of administration showing the empirical 10th, 50th and 90th percentiles and corresponding prediction interval for neutrophil response following 1 mg/kg prednisolone and control administered intravenously (IV, upper row) and after 10 daily doses of 1 mg/kg administered *per os* (PO, lower row) to 9 beagle dogs. Last dose PO was administered at 216 h.

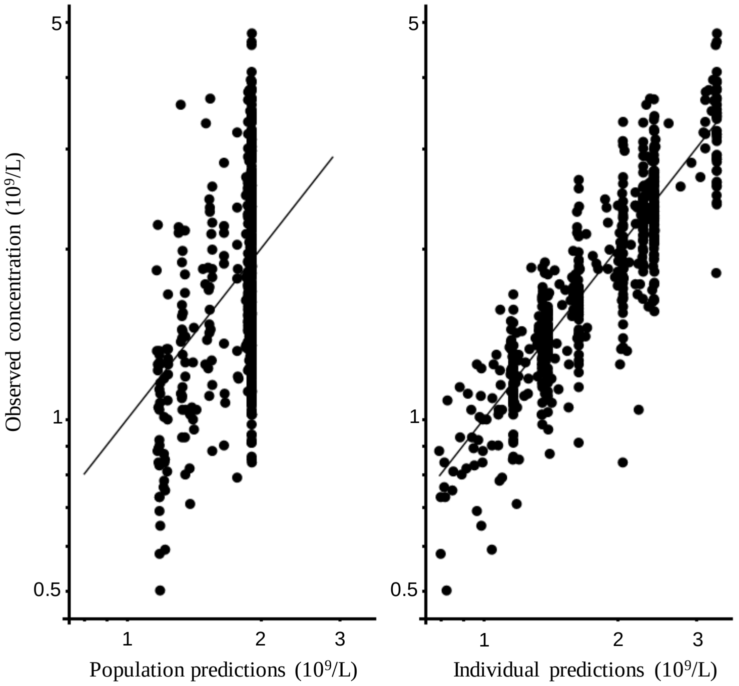

Fig S7. Observed vs predicted lymphocyte whole blood cell count following 1 mg/kg prednisolone administration intravenously (left) and after 10 daily doses of 1 mg/kg administered *per os* (PO, right) to 9 beagle dogs.

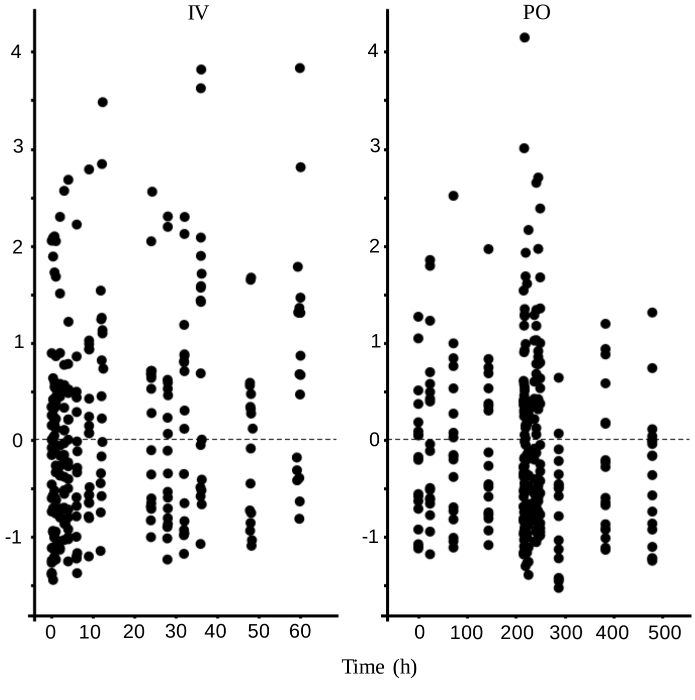

Fig S8. Weighted residuals over time for lymphocyte whole blood cell count splitted by route of administration following 1 mg/kg prednisolone and control administered as a bolus dose intravenously (IV, left) and after 10 daily doses of 1 mg/kg administered *per os* (PO, right) to 9 beagle dogs. Last oral dose was administered at 216 h.

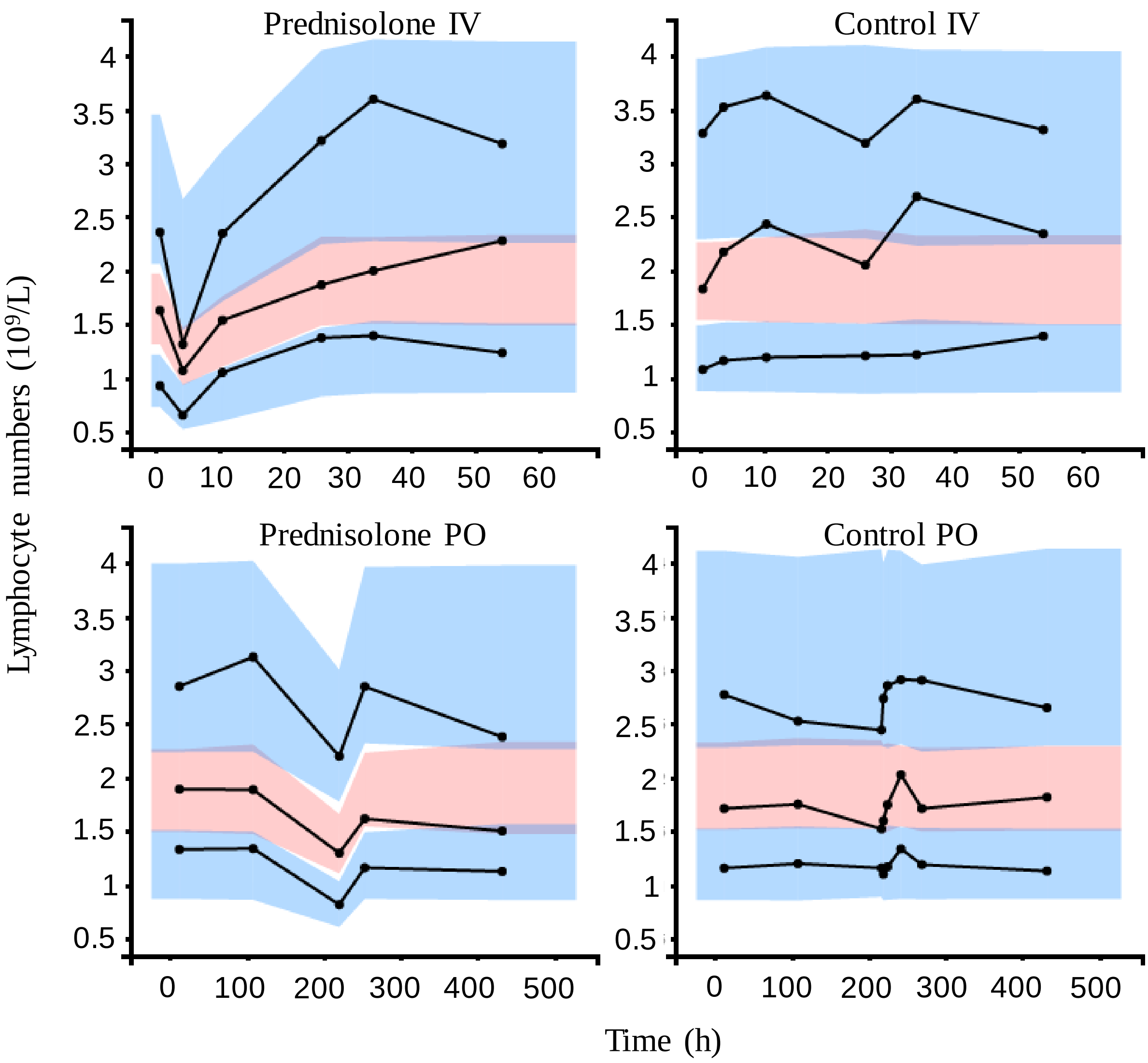

Fig S9. Visual predictive check (VPC) splitted by route of administration showing the empirical 10th, 50th and 90th percentiles and corresponding prediction interval for lymphocyte response following 1 mg/kg prednisolone and control administered intravenously (IV, upper row) and 10 daily doses of 1 mg/kg administered *per os* (PO, lower row) to 9 beagle dogs. Last oral dose was administered at 216 h..

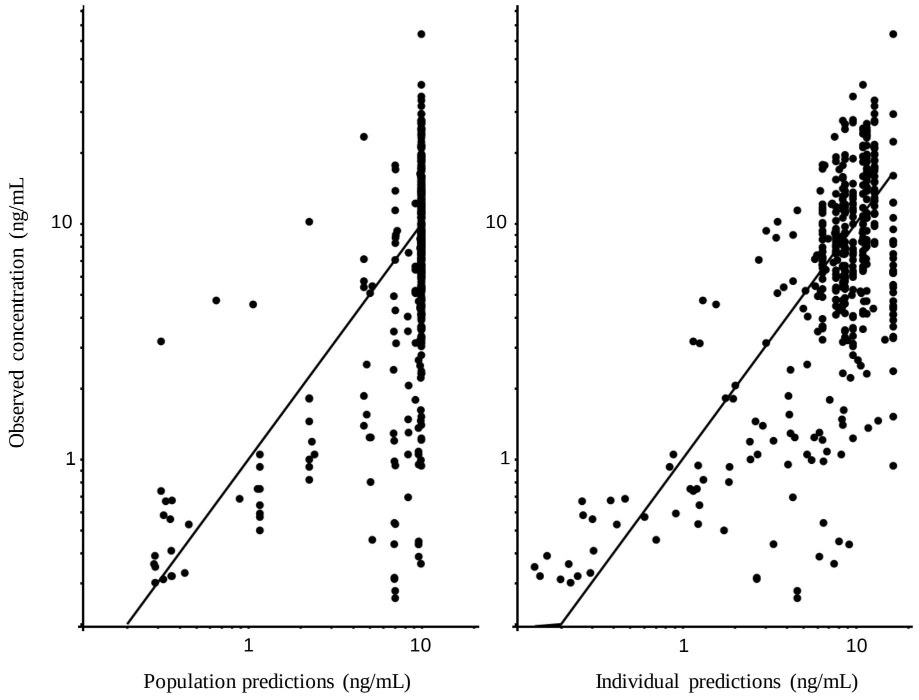

Fig S10. Observed vs predicted cortisol plasma concentration following 1 mg/kg prednisolone administration intravenously (left) and after 10 daily doses of 1 mg/kg administered *per os* (PO, right) to 9 beagle dogs.

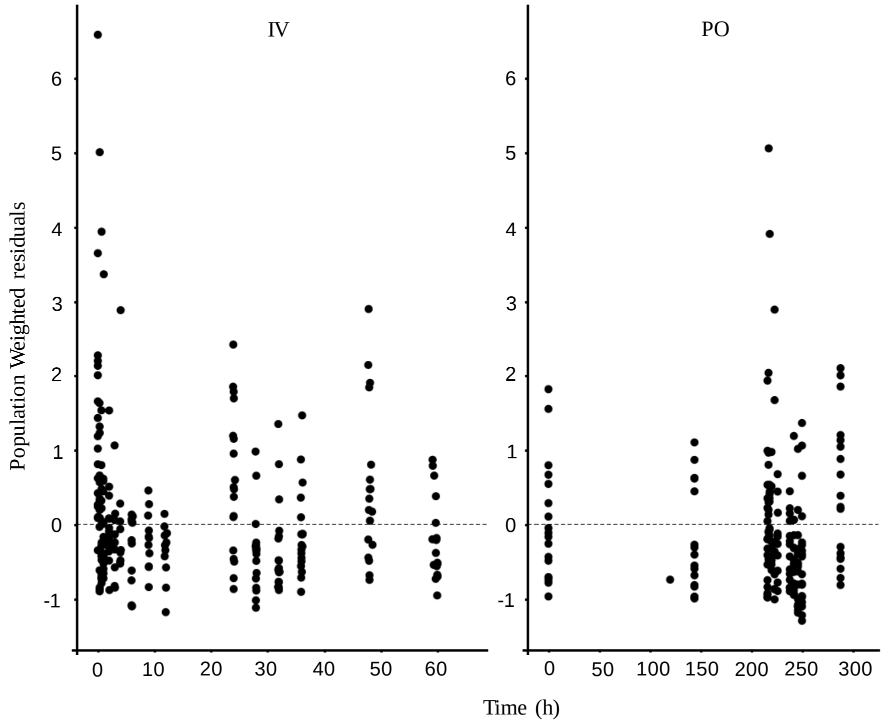

Fig S11. Weighted residuals over time for cortisol plasma concentrations splitted by route of administration following 1 mg/kg prednisolone and control administered intravenously (left) and 10 daily doses of 1 mg/kg administered *per os* (right) to 9 beagle dogs. Last dose was administered at 216 h.

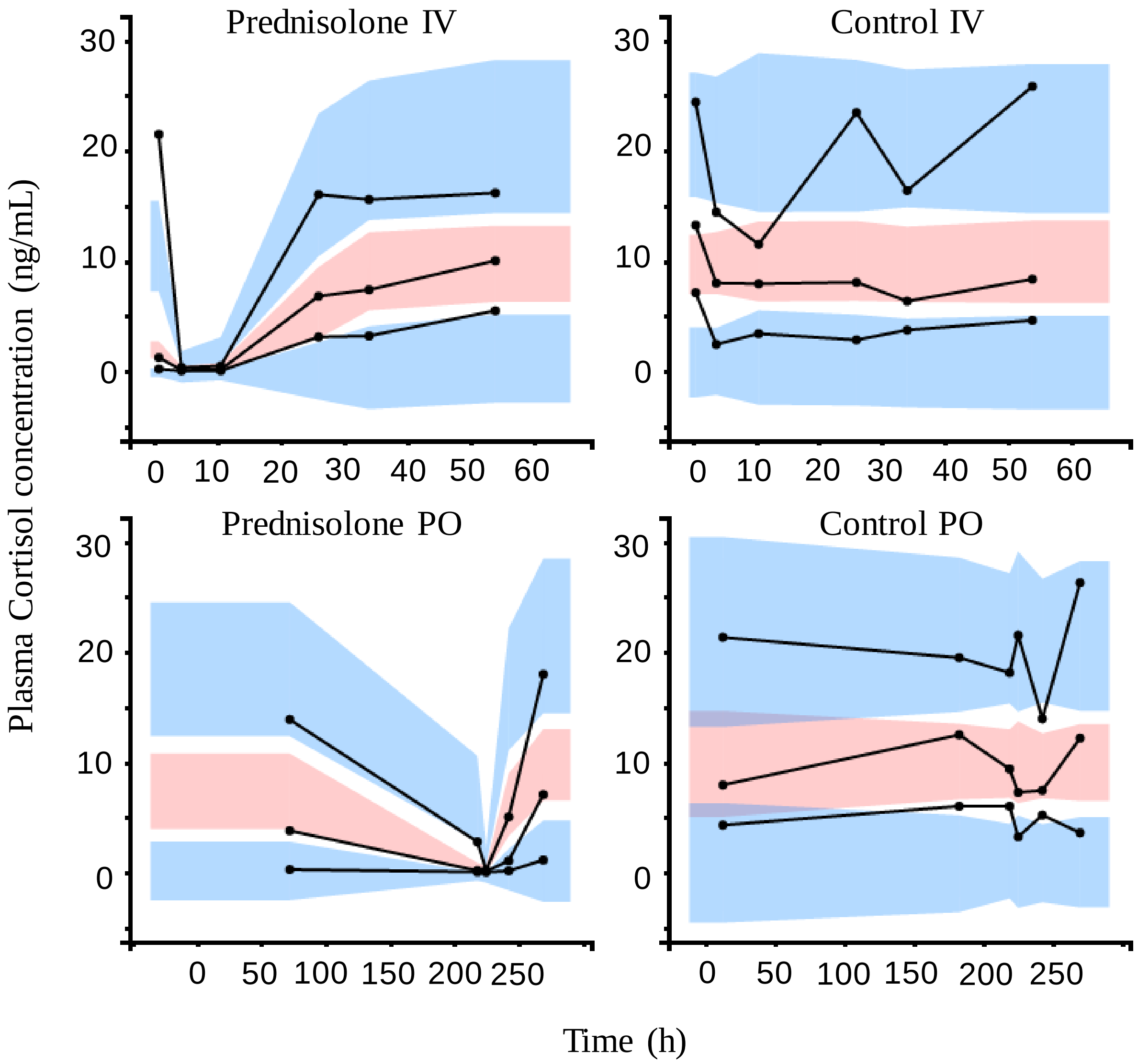

Fig S12. Visual predictive check (VPC) splitted by route of administration showing the empirical 10th, 50th and 90th percentiles and corresponding prediction interval for cortisol response following 1 mg/kg prednisolone and control administered intravenously (IV, upper row) and 10 daily doses of 1 mg/kg administered *per os* (PO, lower row) to 9 beagle dogs. Last oral dose was administered at 216 h.
